# Genetic Relationships, Whole-Fruit Oil Variations, and Quantitative Trait Diversity in Elite *Symplocos paniculata* Accessions from Hunan Revealed by GBS

**DOI:** 10.3390/biology15191702

**Published:** 2026-09-24

**Authors:** Jiating Zhao, Beilei Xie, Lijuan Jiang, Yan Yang, Changzhu Li, Jingzhen Chen, Peiwang Li, Yanju Song, Xiao Zhou, Qiang Liu, Yunzhu Chen

**Affiliations:** 1 State Key Laboratory of Utilization of Woody Oil Resource, Hunan Academy of Forestry, Changsha 410004, China; 2 College of Life Science and Technology, Central South University of Forestry and Technology, 498 South Shaoshan Road, Changsha 410004, China

**Keywords:** *Symplocos paniculata*, genotyping-by-sequencing, genetic diversity, genetic differentiation, ancestry composition, whole-fruit oil content, tissue-specific oil accumulation, fatty acid composition

## Abstract

*Symplocos paniculata* is a promising woody oil species, but the genetic diversity and joint variation in whole-fruit, tissue-specific oil, fatty acid, and quantitative traits remain insufficiently characterized. Thirty elite accessions from six geographic origins in Hunan Province were evaluated using genotyping-by-sequencing, whole-fruit and tissue-specific oil measurements, fatty acid profiling, and 12 quantitative traits. A total of 6335 single-nucleotide polymorphisms revealed geographic differentiation together with substantial within-origin heterogeneity. The principal diversity, PCoA, AMOVA, and pairwise FST patterns remained stable after exclusion of a high-missingness accession. Whole-fruit oil content averaged 34.91% and ranged from 18.54% to 51.35%, with oleic acid as the predominant fatty acid. An additional tissue-specific experiment showed higher mean oil contents in non-seed than seed tissues (37.22% vs. 30.32%) and clear differences in fatty acid composition. Dry-mass-weighted reconstruction closely reproduced measured whole-fruit oil content (Pearson r = 0.935), indicating that whole-fruit variation reflects tissue-specific oil accumulation rather than tissue proportion alone. In the original 72-test analysis, pooled morphology–oil associations did not retain Benjamini–Hochberg significance after adjustment for geographic origin or genetic structure. In a separate 15-test tissue-specific reassessment, infructescence width remained positively associated with non-seed oil content in pooled, origin-adjusted, and genetic-adjusted models. These results characterize complementary genetic and oil-related variations among the sampled accessions and identify tissue-specific relationships that warrant independent validation in replicated common-garden and multi-year trials.

## Simple Summary

*Symplocos paniculata* is a woody oil species whose fruits store oil in both seed and non-seed tissues. We evaluated 30 elite accessions from six geographic origins in Hunan Province using genotyping-by-sequencing, the original whole-fruit measurements, an additional tissue-specific experiment, fatty acid composition, and 12 visible quantitative traits. The accessions showed genetic differentiation among origins together with substantial variation within origins, and the main genetic patterns remained stable after removal of one accession with high genotype missingness. Seed and non-seed tissues differed clearly in both oil content and fatty acid composition, and tissue-weighted reconstruction closely reproduced directly measured whole-fruit oil content. Relationships between visible traits and whole-fruit oil content were weakened after accounting for geographic origin and genetic structure, whereas infructescence width remained positively associated with non-seed oil content in the separate tissue-specific analyses. These findings identify useful variations within the sampled accessions, while also showing that direct chemical measurement and replicated common-garden and multi-year validation are needed before visible traits or regional differences are used for selection or breeding.

## 1. Introduction

*Symplocos paniculata* (Thunb.) Miq. is a deciduous shrub or small tree widely distributed in East and South Asia, with broad ecological adaptability, ornamental value, and potential as a fruit-based woody oil resource [1]. Both the mesocarp and seeds accumulate oil, and the high proportion of unsaturated fatty acids in mature fruits supports its development as a distinctive woody oil crop [2,3,4,5]. Resource development, however, cannot rely on mean oil content alone. It also requires determining whether sampled germplasm represents available genetic variation, whether fatty acid composition is differentiated, and whether associated fruit traits can be evaluated and used consistently under cultivation.

Geographic origin provides a basic framework for germplasm sampling but does not necessarily correspond to genetic populations. In long-lived woody plants, pollen and seed dispersal, local establishment, topographic isolation, and movement of planting material can generate regional differentiation while preserving shared ancestry among locations [6,7,8,9]. Genotyping-by-sequencing can provide large numbers of single-nucleotide polymorphism markers in species lacking a chromosome-level reference genome and thereby resolve such complex genetic patterns [10,11,12]. Genetic information alone, however, does not determine resource value. Whole-fruit oil content and fatty acid composition define chemical quality, whereas leaf, infructescence, and fruit traits describe visible dimensions of vegetative growth, reproduction, and yield formation. Germplasm conservation and pre-breeding therefore require genetic, chemical, and quantitative trait evidence to be considered together.

Previous studies have demonstrated substantial oil accumulation in *S. paniculata* fruit and described the major fatty acids and developmental changes in oil deposition; genetic studies have also detected variations within and among regional populations [2,3,4,5,13,14,15]. These lines of evidence remain fragmented, and genetic relationships, whole-fruit oil traits, and quantitative traits have rarely been analyzed jointly in the same set of elite accessions. A further issue is that pooled correlations in geographically structured material can combine differences among origins with variations among individuals within origins [16,17,18]. Before visible traits are used to prioritize material for oil evaluation, it is necessary to distinguish covariations across the complete sample from relationships that persist after geographic or genetic structure is considered.

This study examined 30 elite *S. paniculata* accessions from six geographic origins in Hunan Province. Filtered genotyping-by-sequencing markers were used to characterize genetic diversity, differentiation, relationships among accessions, and ancestry composition. Whole-fruit oil content and five major fatty acids were quantified, and an additional tissue-specific experiment measured seed and non-seed dry-mass contributions, oil contents, and fatty acid compositions to clarify their contributions to whole-fruit oil variations. The principal patterns of 12 quantitative traits were described, and relationships between quantitative traits and oil-related variables were evaluated in pooled analyses and after adjustment for geographic origin and genetic structure. The robustness of the genetic results was further assessed using locus-filtering and individual-missingness sensitivity analyses. Together, these analyses characterize complementary genetic, chemical, and quantitative trait variations within the sampled accessions and identify candidate relationships for independent validation rather than immediate use as selection criteria.

## 2. Materials and Methods

### 2.1. Plant Materials, Geographic Origins, and Sampling

Thirty *S. paniculata* accessions were sampled from six geographic origins in Hunan Province, China: Changsha (C), Daweishan (D), Daoxian (DX), Hengshan (H), Longshan (L), and Yueyang (Y), with five accessions per origin. Each accession represented one elite tree selected in preceding field surveys. Sampling localities extended from 109.333° to 114.150° E and from 25.400° to 29.367° N at elevations of 78–1550 m. Geographic coordinates, elevations, and Sample IDs are listed in Table 1, and sampling locations are shown in Figure 1A.

Leaves for quantitative trait measurements were collected in July 2021, and mature fruits were collected in October 2021. Leaf and fruit samples were taken from the eastern, southern, western, and northern sectors of each tree canopy to reduce positional variation. Mature fruits were air-dried after collection and stored at 4 °C until whole-fruit oil content and fatty acid composition were measured. Young leaves for genomic DNA extraction were collected in July 2023 from shoots without visible disease or insect damage, wrapped in aluminum foil, immediately frozen in liquid nitrogen, transported to the laboratory, and stored at −80 °C until DNA extraction. The same 30 individually tagged accessions were followed across years. For each accession, the Sample ID assigned during the 2021 phenotypic and chemical sampling was retained for the 2023 DNA sampling; therefore, the genotype, phenotype, and oil-related measurements were linked at the accession level. The Shigongpo germplasm repository shown in Figure 1A was used for ex situ conservation and follow-up maintenance of these accessions and was not treated as an additional geographic origin. An independent batch of mature fruits from the same 30 tagged accessions was collected from September to October during the mature-fruit period for an additional tissue-specific experiment. Fruits were selected after the peel had completed the color transition to a uniform blue to blue-purple coloration, whereas fruits showing visible peel wrinkling or other signs of over-ripening were excluded. This additional experiment was analyzed separately from the original whole-fruit dataset.

### 2.2. Measurement of Leaf, Infructescence, and Fruit Traits

Twelve quantitative traits were measured for each accession. Leaf traits comprised leaf area (LA), leaf length (LL), leaf width (LW), and petiole length (PL). Infructescence traits comprised infructescence length (IL), infructescence width (IW), fruits per infructescence (FPI), and fruit mass per infructescence (FMI). Fruit and reproductive traits comprised fruit longitudinal diameter (FLD), fruit transverse diameter (FTD), 100-fruit fresh weight (HFW), and fruit set (FS).

Three fully developed mature leaves were sampled from one-year-old vegetative shoots in each of the four canopy directions, giving 12 leaves per accession. LL, LW, and PL were measured with a ruler, and LA was measured with a leaf area meter. At fruit maturity, three normally developed infructescences were sampled from each canopy direction, giving 12 infructescences per accession. IL, IW, FPI, and FMI were recorded for these samples. FLD and FTD were measured with a vernier caliper on five normally developed mature fruits. HFW was determined from five independently sampled sets of 100 mature fruits.

Fruit set was calculated from the number of infructescences and the corresponding number of inflorescences recorded on the surveyed branches:

(1) $$FS=\frac{N_{fi}}{N_{inf}}\times 100$$

where *N*_fi_ is the number of infructescences and *N*_inf_ is the corresponding number of inflorescences. The mean of the measurement units was used as the accession-level value for each trait.

### 2.3. Whole-Fruit, Tissue-Specific Oil Content, and Fatty Acid Composition

Mature whole fruits were dried at 105 °C to constant mass, ground, and thoroughly mixed. A 4–6 g portion of whole-fruit powder was wrapped in filter paper and extracted with petroleum ether (boiling range, 30–60 °C) in a Soxhlet apparatus according to GB 5009.6-2016 [19]. Three technical replicates were analyzed for each accession, and their means were used as the accession-level whole-fruit oil content. Whole-fruit oil content was calculated as:

OC = (*m*_o_/*m*_s_) × 100 (2)

where *m*_o_ is the mass of extracted oil, and *m*_s_ is the dry mass of the sample used for extraction.

For the additional tissue-specific experiment, mature fruits meeting the above maturity criteria were analyzed independently from the primary whole-fruit dataset. Within this experiment, FLD and FTD were measured on 10 normally developed mature fruits per accession, and IW was measured on 12 infructescences per accession. For tissue separation, 300 distinct mature fruits were collected from the eastern, southern, western, and northern sectors of each accession canopy. The fruits were combined and randomly allocated into three independent, non-overlapping pools (P1–P3) of 100 fruits each; each fruit was assigned to only one pool. Seeds were removed from each pool, and the non-seed fraction comprised all remaining fruit tissues after seed removal; this fraction was analyzed as a combined non-seed tissue fraction rather than as an isolated mesocarp layer. Seed and non-seed tissues were dried separately at 75 °C to constant mass and weighed individually. Seed and non-seed dry-mass proportions were calculated relative to their combined dry mass:

*P*_seed_ = *m*_seed_/(*m*_seed_ + *m*_nonseed_) × 100 (3)

*P*_nonseed_ = *m*_nonseed_/(*m*_seed_ + *m*_nonseed_) × 100 (4)

where *m*_seed_ and *m*_nonseed_ are the dry masses of seed and non-seed tissues, respectively. The mean of the three independent pools was used as the accession-level tissue dry-mass proportion.

After drying, seed and non-seed tissues were ground separately. For each tissue, 4–6 g of powdered sample was extracted with petroleum ether using a Soxhlet apparatus. Three technical replicates were analyzed for each accession and tissue, and the mean was used as the accession-level tissue oil content. Seed and non-seed oil contents were expressed as the mass of extracted oil relative to the dry mass of the corresponding tissue sample.

Fatty acid composition of whole-fruit oil was determined according to GB 5009.168-2016 [20]. Approximately 60 mg of oil was dissolved in 4 mL of isooctane with gentle heating when required. After the addition of 200 μL of 2 mol/L potassium hydroxide in methanol, the mixture was shaken for 30 s and allowed to clarify, after which approximately 1 g of sodium bisulfate was added. The upper phase was transferred to a gas-chromatography vial and analyzed using a Shimadzu GC-2030 gas chromatograph equipped with a flame ionization detector.

Separation was performed using a DB-23 capillary column (60 m × 0.25 mm × 0.25 μm; Agilent Technologies, Santa Clara, CA, USA). High-purity helium (≥99.999%) was supplied at 1.0 mL/min in constant-flow mode. Injector and detector temperatures were 250 and 280 °C, respectively. The injection volume was 1 μL with a split ratio of 50:1. The oven temperature was initially maintained at 60 °C for 1 min, increased to 175 °C at 25 °C/min and held for 10 min, and then increased to 230 °C at 4 °C/min and held for 5 min. Fatty acid methyl esters were identified by comparison with the retention times of the Supelco 37 Component FAME Mix, and relative abundances were calculated by peak-area normalization.

Seed and non-seed oils were analyzed separately for tissue-specific fatty acid composition after methylation using the same general procedure. The tissue-specific analysis focused on the five major fatty acids detected consistently across all accessions: palmitic acid (C16:0), stearic acid (C18:0), oleic acid (C18:1), linoleic acid (C18:2), and α-linolenic acid (C18:3). Saturated fatty acids (SFA) were calculated as C16:0 + C18:0, monounsaturated fatty acids (MUFA) as C18:1, and polyunsaturated fatty acids (PUFA) as C18:2 + C18:3.

To assess the contribution of tissue composition and tissue-specific oil accumulation to whole-fruit oil content, whole-fruit oil content was reconstructed from the dry-mass proportions and oil contents of the two tissue fractions:

OC_reconstructed_ = *p*_seed_ × OC_seed_ + *p*_nonseed_ × OC_nonseed_ (5)

where *p*_seed_ and *p*_nonseed_ are the seed and non-seed dry-mass proportions expressed as fractions, respectively, and OC_seed_ and OC_nonseed_ are the corresponding tissue oil contents. Whole-fruit oil measurements generated in the additional tissue-specific experiment were used only for within-experiment reconstruction and tissue-specific association analyses and were not pooled with, or used to replace, measurements from the original whole-fruit dataset.

### 2.4. DNA Extraction, GBS Library Preparation, and Primary Bioinformatic Processing

Genomic DNA was extracted from young leaves by a modified CTAB method. DNA concentration was measured with a Qubit fluorometer (Thermo Fisher Scientific, Waltham, MA, USA), and purity was examined with a NanoDrop spectrophotometer (Thermo Fisher Scientific, Waltham, MA, USA).

Qualified DNA was digested with ApeKI, end-repaired, A-tailed, and ligated to Illumina adapters with the NEBNext Ultra DNA Library Prep Kit (New England Biolabs, Ipswich, MA, USA). Fragments of 300–400 bp were selected for PCR amplification and purified with AMPure XP beads (Beckman Coulter, Brea, CA, USA). Library fragment distributions were examined with an Agilent 2100 Bioanalyzer (Agilent Technologies, Santa Clara, CA, USA), and library concentrations were determined by quantitative PCR. Qualified libraries were sequenced as 150 bp paired-end reads on an Illumina NovaSeq 6000 platform (Illumina, San Diego, CA, USA).

Raw reads were filtered with fastp v0.18.0 [21]. Reads containing adapters, reads with at least 10% unknown bases, and reads in which bases with quality scores not exceeding 20 accounted for at least 50% of the sequence were removed. Filtered reads were clustered with Stacks v1.43 [22] using the principal settings m = 6, M = 6, N = 0, and i = 1, with the H option enabled. Overlapping GBS tags were merged, whereas non-overlapping tags were joined with N bases to construct a tag-level pseudo-reference for read alignment and variant detection.

Filtered reads were aligned to the tag-level pseudo-reference with the MEM algorithm in BWA v0.7.12 [23] using k = 32 and the M option. Alignments were processed with Picard v1.129, variants across the accession panel were detected with GATK v3.4-46 [24,25], and variants were annotated with ANNOVAR (version 2) [26]. Filtered-read statistics, tag statistics, variant files, and population genetic analysis results were used in subsequent analyses. Clean reads, clean bases, Q20, Q30, GC content, and the proportion of N bases were used to summarize sequencing yield and base quality. SNPs, InDels, SNP substitution classes, and the transition-to-transversion ratio were used to summarize the primary variant output.

### 2.5. SNP Quality Control and Genetic Diversity Metrics

The SNP genotype table was used as input. Only biallelic SNPs with single-nucleotide reference and alternative alleles were retained. Diploid genotypes were encoded as alternative-allele dosages: 0 for homozygous reference, 1 for heterozygous, and 2 for homozygous alternative genotypes. Missing or non-resolvable genotypes were treated as missing values.

For the reference dataset, sites were retained when the genotype call rate was at least 80%, corresponding to valid genotypes in at least 24 of the 30 accessions; this step retained 8721 SNPs. Minor allele frequency (MAF) was then calculated from non-missing genotypes, and sites with MAF ≥ 0.05 were retained. The final reference dataset therefore contained 6335 SNPs and was used for the primary analyses of genetic diversity, differentiation, genetic relationships, and ancestry composition. The large reduction from raw detected SNPs to call-rate-passing loci occurred primarily at this site-completeness step. Because GBS is a reduced-representation approach and the present analysis used a tag-level pseudo-reference, raw variant discovery can include many loci that are not recovered consistently across accessions because of uneven tag recovery, locus-specific read depths, restriction-site variations, and alignment of tags to the pseudo-reference [10,11,12]. The archived outputs do not permit the relative contribution of these factors to be quantified separately; therefore, the cause of missingness at individual loci was not assigned to a single technical mechanism.

Locus-level sensitivity analysis considered all combinations of call-rate thresholds of 70%, 80%, and 90% and MAF thresholds of 0.03, 0.05, and 0.10. For each combination, the retained SNP count and pairwise genetic distance matrix were recalculated, the corresponding off-diagonal distances were compared with those from the reference filter, and PCoA was repeated for representative liberal, reference, and stringent settings (Figure S4). Only retained SNP counts and pairwise genetic distance matrices were recalculated for all nine locus-filtering combinations; PCoA was repeated for the representative liberal, reference, and stringent settings, whereas diversity indices, AMOVA, pairwise FST, and ADMIXTURE were not recalculated under every locus-filtering combination.

An additional individual-level sensitivity analysis was conducted to evaluate the influence of accessions with high genotype missingness. The accession-level genotype call rate was calculated across the reference 6335-SNP dataset, and an individual call-rate threshold of 70% was used for this sensitivity analysis. This cutoff was used as a targeted sensitivity threshold because it isolated the single clear high-missingness outlier in the panel: L5 had a call rate of 50.69%, whereas the next-lowest accession had a call rate of 71.90%. L5 was therefore excluded for the individual-level sensitivity analysis. The same locus-level filters used for the reference dataset (site call rate ≥ 80% and MAF ≥ 0.05) were then reapplied to the remaining 29 accessions, yielding 5672 SNPs. Genetic distance matrices, PCoA, geographic origin diversity indices, AMOVA, and pairwise FST were recalculated using this sensitivity dataset and compared with the corresponding results from the 30-accession reference dataset.

Observed heterozygosity (Ho), expected heterozygosity (He), unbiased expected heterozygosity (uHe), and polymorphism information content (PIC) were calculated separately for each geographic origin. In the reference dataset, a locus was included in an origin-level estimate when at least three of the five accessions from that origin had valid genotypes. For a biallelic locus with allele frequencies p and q, He was calculated as 2pq, and PIC was calculated as 1 − (p^2^ + q^2^) − 2p^2^q^2^, which is equivalent to 2pq (1 − pq). Origin-level values were obtained by averaging across retained loci. In the individual-level sensitivity dataset, the same calculation was applied, with the retained Longshan group containing four accessions after exclusion of L5.

A one-level analysis of molecular variance (AMOVA) [27] partitioned genetic variation among and within the six geographic origins. AMOVA used Euclidean distances between genotype-dosage vectors normalized by the number of loci with valid genotypes for each accession pair. Significance of ΦST in the reference analysis was assessed with 9999 permutations using the random seed 20260823. Pairwise FST values were calculated with the Weir and Cockerham estimator [28]. For each origin pair in the reference dataset, the exact randomization distribution comprised the 126 unique allocations of the ten accessions into two groups of five. The 15 exact p-values were adjusted together using the Benjamini–Hochberg procedure. For the individual-level sensitivity dataset, pairwise FST estimates were recalculated with the same Weir–Cockerham estimator and compared directly with the reference estimates. Because the archived run-level records did not preserve the exact two-sided randomization convention required for a directly comparable sensitivity p-value workflow, exact sensitivity p- and q-values were not interpreted.

### 2.6. Genetic Relationships and Ancestry Composition

For each accession pair, genetic distance was calculated across loci with valid genotypes in both accessions. At each locus, the absolute difference in alternative-allele dosage was divided by two and averaged across shared non-missing loci:

d_ij_ = (1/|L_ij_|) Σ_l_∈L_ij_ |g_il_ − g_jl_|/2

where L_ij_ is the set of loci with valid genotypes in accessions i and j, and g_il_ and g_jl_ are their alternative-allele dosages at locus l. Classical principal coordinate analysis (PCoA) [29] was applied to the resulting genetic distance matrix. Cailliez additive correction [30] was used to address negative eigenvalues, and the proportion explained by each axis was calculated from the corresponding positive eigenvalue divided by the sum of all positive eigenvalues. The same distance calculation and PCoA procedure were applied to the 29-accession individual-missingness sensitivity dataset described in Section 2.5.

The neighbor-joining tree was obtained from the TreeBeST result and interpreted according to neighbor-joining topology [31]. Tree topology and internal-node bootstrap support values [32] were extracted from the archived NHX file and redrawn as a rectangular cladogram; branch lengths in the redrawn panel were used only for topological layout.

Ancestry composition was summarized from the archived ADMIXTURE outputs available for K = 1–9 [33,34]. The corresponding cross-validation curve was used to compare the supplied K solutions. K = 2 was used to illustrate the coarse-scale ancestry composition. K = 8 corresponded to the minimum cross-validation error in the available outputs and was therefore shown only as an exploratory finer-scale representation rather than as a definitive estimate of the number of biological populations.

The archived ADMIXTURE result set did not contain run-level information sufficient to verify whether linkage-disequilibrium pruning had been applied or whether independent replicate runs had been used to assess convergence and cluster stability. Accordingly, ADMIXTURE was interpreted descriptively and was not used as the sole basis for delineation of discrete genetic groups or for inference of historical gene flow. Interpretation of genetic structure was based jointly on PCoA, the neighbor-joining topology, genetic diversity indices, AMOVA, pairwise FST, and the descriptive ancestry profiles. ADMIXTURE was not rerun for the 29-accession L5-exclusion sensitivity dataset because the archived files did not preserve the preprocessing and replicate-run information needed to reproduce the original ADMIXTURE workflow exactly. Accordingly, the robustness conclusions from the individual-level sensitivity analysis are based on PCoA, pairwise genetic distances, origin-level diversity indices, AMOVA, and pairwise FST, and do not include an ADMIXTURE sensitivity claim.

### 2.7. Statistical Analysis of Oil, Fatty Acids, Tissue-Specific Traits, and Quantitative Traits

For whole-fruit oil content, fatty acid composition, tissue-specific measurements, and the 12 quantitative traits, accession-level values were used for statistical analysis. The mean, standard deviation, median, first and third quartiles, minimum, maximum, and coefficient of variation were calculated as appropriate. Because each geographic origin contained five accessions, comparisons among the six origins used the Kruskal–Wallis test [35].

Whole-fruit oil content was first tested across the six geographic origins. When the global test was significant, Dunn pairwise comparisons were performed [36], and the 15 pairwise *p*-values were adjusted together using the Benjamini–Hochberg procedure [37]. The five major fatty acids were tested separately with Kruskal–Wallis tests, and the five global *p*-values were adjusted as one family. Saturated fatty acids (SFA), monounsaturated fatty acids (MUFA), and polyunsaturated fatty acids (PUFA) were analyzed in the same manner, with the three global *p*-values adjusted as a separate family. The 12 quantitative traits were tested separately, and the 12 global *p*-values were adjusted together.

For the additional tissue-specific experiment, seed and non-seed dry-mass proportions, tissue oil contents, and tissue fatty acid compositions were summarized across the 30 accessions. Because seed and non-seed measurements were obtained from the same accession, seed and non-seed oil contents were compared using a paired Wilcoxon signed-rank test. The five major fatty acids (C16:0, C18:0, C18:1, C18:2, and C18:3) were likewise compared between seed and non-seed tissues using paired Wilcoxon signed-rank tests, and the five resulting *p*-values were adjusted together using the Benjamini–Hochberg procedure.

Agreement between measured and reconstructed whole-fruit oil content was evaluated across the 30 accessions using Pearson and Spearman correlations. The measured-minus-reconstructed paired difference was additionally summarized together with the mean absolute error (MAE) and root mean square error (RMSE) to quantify reconstruction bias and error. The mean measured-minus-reconstructed difference was tested against zero using a two-sided paired *t*-test.

The five major fatty acids constituted compositional data. Their relative abundances were first renormalized to sum to one within each accession and then transformed using the centered log-ratio (CLR) transformation [38,39]:

clr(p_ij_) = ln[p_ij_/(∏_k_ = _1_^5^ p_ik_)^(1/5)]

where p_ij_ is the renormalized proportion of fatty acid j in accession i. All five values were positive, so zero replacement was unnecessary. For graphical summaries, the CLR deviation for an origin was defined as the origin mean minus the overall mean across all 30 accessions. Descriptive 95% intervals were calculated from the origin-specific standard error with four degrees of freedom and were used to display direction and within-origin variability.

Quantitative traits were standardized across the 30 accessions as Z scores for comparisons among origins. Unless otherwise stated, all tests were two-sided. Unadjusted single tests were interpreted at *p* < 0.05, and predefined multiple-testing families were interpreted at Benjamini–Hochberg-adjusted q < 0.05.

Principal component analysis [40] was performed on the standardized matrix of 12 quantitative traits. The biplot displayed accession scores and trait-loading vectors. Convex hulls were drawn around accessions from the same geographic origin to show their observed ranges in the first two principal component dimensions and were treated as descriptive boundaries rather than confidence regions.

### 2.8. Relationships Between Quantitative Traits and Oil-Related Variables

Relationships between the 12 quantitative traits and six oil-related variables were first examined across all 30 accessions. The six oil-related variables comprised whole-fruit oil content and the centered log-ratio (CLR) values of C16:0, C18:0, C18:1, C18:2, and C18:3, giving 72 trait-by-variable combinations. Spearman rank correlation was calculated for each combination across the complete set of accessions, and the 72 *p*-values were adjusted together using the Benjamini–Hochberg procedure.

Geographic origin was then included in a separate structure-adjusted analysis. For each quantitative trait and oil-related variable, values were converted to ranks and separately regressed on five dummy variables representing the six geographic origins. Pearson correlation was calculated between the corresponding residuals. The 72 *p*-values from this origin-adjusted analysis constituted a separate Benjamini–Hochberg family.

Genetic structure was evaluated in a third analysis using PCoA1 and PCoA2 obtained from the final 6335-SNP distance matrix. Each quantitative trait rank and oil-variable rank was separately regressed on PCoA1 and PCoA2, followed by Pearson correlation between the corresponding residuals. The 72 *p*-values from this genetic-adjusted analysis constituted another separate Benjamini–Hochberg family.

For the 12 origin-adjusted relationships involving whole-fruit oil content, uncertainty was further evaluated using 10,000 stratified bootstrap replicates [41]. In each replicate, accessions were sampled with replacement within each of the six geographic origins while retaining five accessions per origin, and the adjusted coefficient was recalculated. The 2.5th and 97.5th percentiles of the bootstrap distribution were used as the 95% confidence interval.

The additional tissue-specific experiment was analyzed independently to reassess the morphology–oil relationships emphasized in the primary whole-fruit analysis. The fruit longitudinal diameter (FLD), fruit transverse diameter (FTD), infructescence width (IW), and measured whole-fruit oil content used in this reassessment were all obtained from the same experimental batch used for tissue separation rather than from the primary dataset. FLD, FTD, and IW were evaluated against five tissue- or oil-related variables: measured whole-fruit oil content, seed dry-mass proportion, seed oil content, non-seed oil content, and reconstructed whole-fruit oil content. The resulting design comprised 15 morphology-by-variable combinations.

For this 15-test tissue-specific reassessment, pooled associations were evaluated using Spearman rank correlations across all 30 accessions. Origin-adjusted associations were evaluated by rank-transforming each variable, separately residualizing the ranked values against the same five geographic origin dummy variables described above, and calculating Pearson correlations between the resulting residuals. Genetic-adjusted associations were evaluated in the same manner after residualizing ranked variables against PCoA1 and PCoA2 from the final 6335-SNP distance matrix. The 15 *p*-values within each of the pooled, origin-adjusted, and genetic-adjusted analyses were treated as three separate multiple-testing families and were adjusted independently using the Benjamini–Hochberg procedure.

The original 72-test analyses and the tissue-specific 15-test reassessment were interpreted separately because they addressed different sets of biological variables and constituted different predefined multiple-testing families. Statistical significance after multiple-testing correction was defined as Benjamini–Hochberg-adjusted q < 0.05; consistency in coefficient direction across analytical models was described separately from statistical significance.

### 2.9. Software and Reproducibility

Statistical analyses were performed in R v4.3.2 and Python v3.11. Principal component analysis used prcomp in the R stats package. Kruskal–Wallis tests, Spearman correlations, and Benjamini–Hochberg adjustments used kruskal.test, cor.test, and p.adjust, respectively; Dunn pairwise comparisons used rstatix v0.7.2. Data handling and graphical output used ggplot2 v3.4.4 [42], dplyr v1.1.4, readr v2.1.5, readxl v1.4.3, svglite v2.1.3, ragg v1.2.7, pandas v2.1.4, NumPy v1.26.4, SciPy v1.11.4, Matplotlib v3.8.2, and CairoSVG v2.7.1. SNP filtering, origin-level diversity statistics, AMOVA, pairwise FST, adjusted correlation analyses, and bootstrap calculations were performed with reproducible scripts and fixed random seeds where applicable.

Genetic, whole-fruit oil, fatty acid, and quantitative trait data were organized using analysis Sample IDs 1–30. Origin-coded Figure IDs (C1–C5, D1–D5, DX1–DX5, H1–H5, L1–L5, and Y1–Y5) were used when individual accessions were labeled in the main and supplementary figures; their one-to-one correspondence with analysis Sample IDs is provided in Supplementary Table S1. Accession-level measurements, sequencing-quality statistics, and numerical values underlying the main figures are provided in Supplementary Tables S1–S11.

## 3. Results

### 3.1. Sampling Framework and GBS Data Quality

The 30 *S. paniculata* accessions represented six geographic origins in Hunan Province, with five accessions per origin (Table 1; Figure 1A). Sampling localities extended from 109.333° to 114.150° E and from 25.400° to 29.367° N, at elevations of 78–1550 m.

GBS generated 95.55 Gb of raw sequence data, of which 95.20 Gb remained after read filtering. A total of 671,519,072 clean reads were retained, averaging 3.17 Gb per accession. The mean GBS tag length was 142 bp. The accession-level clean-data volume, Q30, and genotype missingness in the filtered SNP matrix are shown in Figure 1B. The GC content ranged from 38.18% to 39.52%, and the mean Q20 and Q30 values were 97.47% and 92.73%, respectively.

Primary variant detection yielded 1,971,135 loci, including 1,935,147 SNPs and 35,988 InDels, which represented 98.17% and 1.83% of all detected variants, respectively. Among the SNPs, 1,377,923 were transitions, and 557,224 were transversions, corresponding to 71.21% and 28.79%, and a transition-to-transversion ratio of 2.47. C-to-T and G-to-A substitutions were the two most frequent classes, representing 20.59% and 20.39% of the SNPs, respectively (Figure 1C).

Of the 1,935,147 raw detected SNPs, 8721 had genotype call rates of at least 80%. After retaining sites with minor allele frequencies of at least 0.05, 6335 SNPs remained for subsequent genetic analyses of the 30 accessions (Figure 1C). Across all nine alternative call-rate and MAF combinations, correlations between the recalculated genetic distance matrices and the reference matrix ranged from 0.961 to 0.999. PCoA was repeated only for the representative liberal, reference, and stringent filtering settings, under which the major ordination pattern was retained (Figure S4). The call-rate criterion was therefore the dominant filtering step: 8721 of 1,935,147 raw detected SNPs (0.45%) passed the ≥80% site call-rate threshold, after which the MAF criterion removed a further 2386 loci (27.36% of the call-rate-passing SNPs).

### 3.2. Genetic Diversity, Differentiation, and Ancestry Patterns

Origin-level diversity indices varied among the six sampled geographic origins (Table 2). Among the sampled accessions, Longshan had the highest Ho (0.115), He (0.225), and PIC (0.180), whereas Daweishan had the lowest corresponding values (Ho = 0.024, He = 0.099, and PIC = 0.078). Ho was lower than He in every origin. The remaining origins had He values of 0.126–0.153 and PIC values of 0.098–0.119.

AMOVA attributed 33.38% of the genetic variation to differences among geographic origins and 66.62% to variation within origins. The overall differentiation estimate was ΦST = 0.3338, with a permutation *p*-value of 0.0001 based on 9999 permutations (Figure 2E). Pairwise FST estimates for the 15 origin pairs ranged from −0.005 to 0.568, with Changsha versus Daoxian being the lowest and Daweishan versus Yueyang the highest. Eight pairwise comparisons remained significant after Benjamini–Hochberg correction. The largest significant estimates were observed for Daweishan versus Yueyang (0.568), Changsha versus Daweishan (0.556), and Daweishan versus Daoxian (0.555).

In the Cailliez-corrected PCoA, the first and second coordinates explained 47.51% and 16.37% of the positive-eigenvalue variation, respectively (Figure 2A). The 30 accessions formed a partly continuous distribution rather than six discrete, non-overlapping groups corresponding to collecting origin. Some accessions from the same origin were close in ordination space, whereas others were separated along the first two coordinates, and accessions from different origins overlapped in several regions. The neighbor-joining tree showed a similar pattern (Figure 2B): several terminal groups contained accessions from the same origin, but major branches included accessions from multiple origins, and accessions assigned to the same origin occurred on different branches.

The archived ADMIXTURE analysis included solutions for K = 1–9 and a corresponding cross-validation curve. The cross-validation error reached its minimum at K = 8 (Figure 2C). At K = 2, both ancestry components were represented across multiple geographic origins, illustrating coarse-scale shared ancestry. The K = 8 solution showed finer-scale heterogeneity among accessions, including variation within origins (Figure 2D). Because the archived analysis did not include run-level records sufficient to verify LD pruning or stability across independent replicate runs, the K = 8 pattern was treated as an exploratory finer-scale representation rather than as evidence for a definitive number of genetic populations.

Individual-level sensitivity analysis identified L5 as the only accession with a genotype call rate below 70% in the reference 6335-SNP dataset (50.69%; the next-lowest call rate was 71.90%). After excluding L5 and reapplying the same locus filters, 5672 SNPs were retained for 29 accessions. The sensitivity PCoA remained highly concordant with the reference ordination: PCoA1 and PCoA2 explained 46.52% and 16.77% of the positive-eigenvalue variation, respectively. For the 29 accessions shared by both analyses, the corresponding PCoA1 and PCoA2 coordinates were correlated at r = 0.99984 and r = 0.99956, respectively, and the pairwise genetic distance matrices were correlated at r = 0.99968 (Figure S7; Table S11).

The principal diversity and differentiation results were also retained after exclusion of L5 (Table S11). Longshan, represented by four accessions in the sensitivity dataset, retained the highest He (0.224) and PIC (0.178) among the sampled origins. AMOVA assigned 34.91% of the variation to differences among origins and 65.09% to the variation within origins (ΦST = 0.3491, *p* = 0.0001), closely matching the reference result. Pairwise FST estimates in the sensitivity dataset ranged from −0.0087 to 0.5597, and the absolute changes relative to the corresponding reference estimates did not exceed 0.0393. Thus, the major ordination, diversity, and differentiation patterns were not driven by the high-missingness accession. Because the original ADMIXTURE preprocessing and replicate-run settings could not be reconstructed exactly from the archived files, ADMIXTURE was not included in the L5-exclusion sensitivity comparison, and no robustness claim for ADMIXTURE was made.

### 3.3. Variation in Whole-Fruit Oil Content and Fatty Acid Composition

Whole-fruit oil content ranged from 18.54% to 51.35% across the 30 accessions, with a mean of 34.91 ± 9.49%, a median of 34.42%, and a coefficient of variation of 27.18% (Table 3; Figure 3A). Mean oil content by geographic origin was 43.63% for Daweishan, 42.53% for Longshan, 36.55% for Hengshan, 30.45% for Daoxian, 29.16% for Changsha, and 27.14% for Yueyang. The global Kruskal–Wallis test indicated a significant difference among origins (*p* = 0.0143; Figure 3A).

The five major fatty acids were C16:0, C18:0, C18:1, C18:2, and C18:3 (Figure 3B). Their mean relative abundances were 26.24 ± 3.41%, 1.03 ± 0.17%, 48.53 ± 6.56%, 22.01 ± 5.64%, and 1.46 ± 0.43%, with ranges of 21.06–33.54%, 0.80–1.60%, 38.47–63.16%, 10.97–31.71%, and 0.76–2.65%, respectively. C18:1, C18:2, and C18:3 together accounted for 72.00% of the whole-fruit fatty acid composition.

The mean SFA, MUFA, and PUFA values were 27.28%, 48.53%, and 23.47%, with ranges of 22.12–34.47%, 38.47–63.17%, and 12.57–33.78%, respectively. Their distributions among geographic origins are shown in Figure 3D, and accession-level values with origin means are shown in Figure S1. The Kruskal–Wallis tests gave H = 12.649, 10.012, and 9.810 and unadjusted *p* = 0.0269, 0.0749, and 0.0808 for SFA, MUFA, and PUFA, respectively. After Benjamini–Hochberg adjustment within the three-test family, the corresponding q-values were 0.0807, 0.0808, and 0.0808, and none reached q < 0.05 (Table 3).

The CLR summaries described the direction and uncertainty of origin-level compositional deviations (Figure 3C). Daweishan had a positive C16:0 CLR deviation of 0.207, with a descriptive 95% interval of 0.118–0.296 around the origin mean. Deviations for the other fatty acids and origins included both positive and negative values, with several intervals spanning the overall compositional center.

### 3.4. Tissue-Specific Oil Content and Fatty Acid Composition

In the additional tissue-specific experiment, the mean seed dry-mass proportion across the 30 accessions was 46.62 ± 2.90%, whereas the corresponding non-seed proportion was 53.38 ± 2.90% (Figure S5A; Table S9). The seed oil content averaged 30.32 ± 1.91%, compared with 37.22 ± 3.49% in non-seed tissues. The non-seed oil content exceeded the seed oil content in 27 of the 30 accessions, with a mean paired difference of 6.90 percentage points. The paired Wilcoxon signed-rank test indicated a significant difference between the two tissue fractions (*p* < 0.001; Figure S5B).

The five major fatty acids also showed clear tissue-specific differences (Figure S5D; Table S9). Seed oil contained higher mean proportions of C18:1 and C18:2 than non-seed oil at 52.35% versus 38.27% and 31.79% versus 23.83%, respectively. In contrast, non-seed oil contained higher mean proportions of C16:0, C18:0, and C18:3, at 33.30%, 1.52%, and 1.95%, compared with 12.76%, 1.30%, and 1.02% in seed oil. All five tissue comparisons remained significant after Benjamini–Hochberg correction within the five-test family (q < 0.05).

Dry-mass-weighted reconstruction closely reproduced the directly measured whole-fruit oil content (Figure S5C). The reconstructed whole-fruit oil content averaged 34.02 ± 1.95%, compared with 33.98 ± 2.03% for the corresponding direct whole-fruit measurements obtained within the same additional experiment. The two estimates were strongly correlated (Pearson r = 0.935, *p* < 0.001; Spearman ρ = 0.921, *p* < 0.001). The mean measured-minus-reconstructed difference was −0.046 percentage points and did not differ significantly from zero (paired *t*-test, *p* = 0.731). The mean absolute error and root mean square error were 0.594 and 0.712 percentage points, respectively, indicating close agreement between the direct and tissue-weighted estimates.

### 3.5. Quantitative Trait Variations and Multivariate Phenotypic Structures

All 12 quantitative traits varied among the 30 accessions (Table 3; Figure 4B). FS and IW had the largest coefficients of variation, at 48.18% and 47.97%, respectively. FPI, LA, and FMI had coefficients of variation of 32.80%, 32.14%, and 31.39%, respectively. FTD, LW, and FLD showed a comparatively lower relative variation.

Principal component analysis summarized the joint trait variation (Figure 4A). PC1 and PC2 explained 35.2% and 23.8% of the standardized trait variation, respectively. Positive PC1 scores were associated mainly with leaf size, petiole length, and fruit diameters, whereas negative PC1 scores were associated mainly with infructescence length, FPI, and FMI. PC2 separated accessions with higher leaf-size and HFW loadings from accessions with lower scores along this axis. Origin-specific convex hulls overlapped in several regions of the biplot.

Figure 4C shows the median and interquartile ranges of the Z scores for all 12 traits in each geographic origin. After joint adjustment of the 12 Kruskal–Wallis tests, seven traits differed among origins at q < 0.05: IW and FLD each had q = 0.0104, FTD and LL each had q = 0.0196, LW had q = 0.0351, PL had q = 0.0389, and LA had q = 0.0413. Detailed origin-specific distributions and the corresponding H-, *p*-, and q-values for all 12 traits are shown in Figure S2.

### 3.6. Relationships Between Quantitative Traits and Oil-Related Variables Before and After Adjustment for Origin and Genetic Structure

In the original pooled analysis across all 30 accessions, 9 of the 72 relationships between the 12 quantitative traits and six oil-related variables remained significant after Benjamini–Hochberg correction (Figure 4D; Table S7). Five of these involved whole-fruit oil content. Infructescence width (IW), fruit transverse diameter (FTD), and fruit longitudinal diameter (FLD) showed the strongest positive pooled relationships with whole-fruit oil content, with Spearman ρ values of 0.636, 0.635, and 0.632, respectively. Petiole length and leaf length were also positively related to whole-fruit oil content in the pooled analysis, with ρ = 0.563 and 0.510. Four additional pooled relationships that remained significant after correction involved fatty acid CLR values.

Adjustment for geographic origin substantially reduced the coefficients of several pooled morphology–oil relationships. For IW, FTD, and FLD versus whole-fruit oil content, the origin-adjusted residual correlations were 0.372, 0.270, and 0.189, respectively. After adjustment for PCoA1 and PCoA2, the corresponding residual correlations were 0.503, 0.511, and 0.485. Although the directions of these coefficients remained positive, none of the 72 relationships in either the origin-adjusted or genetic-adjusted analyses remained significant after Benjamini–Hochberg correction within the corresponding 72-test family. The origin-stratified bootstrap analysis likewise showed substantial uncertainty around the adjusted whole-fruit-oil relationships (Figure S3). Thus, persistence of coefficient direction across models was distinguished from multiplicity-corrected statistical support.

The additional tissue-specific experiment was analyzed separately for the 15 combinations of FLD, FTD, and IW with measured whole-fruit oil content, seed dry-mass proportion, seed oil content, non-seed oil content, and reconstructed whole-fruit oil content (Figure S6; Table S10). In the pooled analysis, IW was positively associated with non-seed oil content (Spearman ρ = 0.555, *p* = 0.00145, q = 0.0218), and this was the only relationship that remained significant after correction within the pooled 15-test family. Pooled relationships of FLD, FTD, and IW with seed dry-mass proportion were weak and non-significant, as were their relationships with seed oil content after multiple-testing correction.

After adjustment for geographic origin, the relationship between IW and non-seed oil content remained positive and significant (residual r = 0.570, *p* = 0.00102, q = 0.0153). The same relationship also remained significant after adjustment for PCoA1 and PCoA2 (residual r = 0.556, *p* = 0.00141, q = 0.0211). Several relationships involving measured or reconstructed whole-fruit oil content were also significant within the origin-adjusted or genetic-adjusted 15-test families (Table S10), whereas seed dry-mass proportion and seed oil content showed no significant relationships with FLD, FTD, or IW in any of the three models.

Because the tissue-specific analysis involved a different set of variables and a separate 15-test multiple-testing family, its adjusted q-values were interpreted independently from those of the original 72-test analysis. Across the three tissue-specific models, the IW-non-seed oil relationship showed the most consistent multiplicity-corrected statistical support, whereas the original whole-fruit trait associations were treated as candidate relationships whose directions were informative but whose statistical support depended on the analytical framework.

## 4. Discussion

### 4.1. Genetic Differentiation and Within-Origin Heterogeneity Among the Sampled Accessions

The 30 elite *S. paniculata* accessions showed measurable genetic differentiation among the six sampled geographic origins, together with substantial variation within origins. AMOVA attributed 33.38% of the total genetic variation to differences among origins and 66.62% to variation within origins, while pairwise FST estimates ranged from values close to zero to 0.568. The PCoA and neighbor-joining tree likewise showed partial geographic clustering without complete separation of accessions by origin. These results indicate that geographic origin captured part of the genetic structure in the sampled material, but did not define six discrete genetic populations.

Several biological processes could generate the observed combination of differentiation and overlap. Spatial isolation in mountainous environments may increase differentiation, whereas pollen dispersal, animal-mediated seed movement, or movement of plant material can reduce differentiation among locations [43,44]. However, the present sampling design does not directly distinguish among these processes or reconstruct historical gene flow. The ADMIXTURE outputs are therefore interpreted descriptively. The K = 2 solution illustrates coarse-scale shared ancestry across several origins, whereas K = 8, which corresponded to the minimum of the archived cross-validation curve, is treated only as an exploratory finer-scale representation because the archived files were insufficient to verify LD pruning or stability across independent replicate runs.

The individual-level missingness sensitivity analysis further indicated that the main genetic conclusions were not driven by the high-missingness accession L5. After excluding L5 and reapplying the same locus-level filters, 5672 SNPs were retained for 29 accessions. The resulting PCoA was nearly identical to the reference ordination, the pairwise genetic distance matrix was highly correlated with that of the reference dataset, and AMOVA produced a similar partition of variation (34.91% among origins and 65.09% within origins). Pairwise FST magnitudes also changed only modestly, with an absolute difference of no more than 0.0393 across the 15 origin pairs. Longshan retained the highest He and PIC among the sampled origin groups after L5 was excluded. Together, these results support the robustness of the principal diversity and differentiation patterns to this source of genotype missingness. The sharp raw-to-filtered SNP reduction was driven mainly by the site call-rate criterion. The locus-threshold sensitivity analysis demonstrated high concordance of genetic distance matrices across all nine filter combinations (Figure S4). PCoA was repeated only for representative liberal, reference, and stringent filtering settings and retained the major ordination pattern under those settings; diversity indices, AMOVA, pairwise FST, and ADMIXTURE were not recalculated under every locus-filtering combination. The L5 sensitivity conclusions likewise exclude ADMIXTURE because an exactly reproducible rerun could not be guaranteed from the archived ADMIXTURE workflow.

Genetic diversity nevertheless varied among the sampled origin groups, with Longshan showing comparatively high diversity and Daweishan lower values in the present panel. Such differences should be interpreted cautiously because only five elite accessions were sampled from each origin in the reference analysis, and the materials were selected rather than randomly sampled from natural populations. Relatedness among accessions, local demographic history, and the initial selection of elite trees may influence the observed diversity estimates [45]. Broader sampling within and beyond these six geographic origins will therefore be required to determine how representative the present patterns are across the natural distribution of *S. paniculata*.

### 4.2. Whole-Fruit Oil Variation Reflects Tissue-Specific Oil Accumulation and Fatty Acid Composition

Whole-fruit oil content and fatty acid composition showed substantial variations among the 30 accessions, consistent with previous reports that both seed and non-seed tissues contribute to oil accumulation in *S. paniculata* fruit [2,3,4,5]. In the original whole-fruit measurements, the oil content averaged 34.91% and ranged from 18.54% to 51.35%, while C18:1 was the predominant fatty acid, and unsaturated fatty acids accounted for approximately 72% of the measured profile. These whole-fruit values provide an integrated measure of fruit oil quantity and composition, but they do not by themselves distinguish variations in tissue proportion from variations in tissue-specific oil concentration.

Plant oil accumulation depends on coordinated fatty acid synthesis and triacylglycerol assembly, and oil deposition in storage tissues is developmentally regulated [46,47]. The additional tissue-specific experiment helps resolve this issue. Across the 30 accessions, non-seed tissues had a higher mean oil content than seeds, and the non-seed oil content exceeded the seed oil content in 27 accessions. The two tissue fractions also differed markedly in fatty acid composition: seed oil contained higher proportions of C18:1 and C18:2, whereas non-seed oil contained a much higher proportion of C16:0 together with higher C18:0 and C18:3. These results are consistent with previous evidence that oil-rich fruit tissues and seeds can differ in carbon partitioning and lipid biosynthesis [48,49,50], and they show that whole-fruit fatty acid composition represents a weighted mixture of chemically distinct tissue fractions rather than a uniform oil pool.

Importantly, tissue proportion alone did not account for the observed whole-fruit oil variation. Within the additional tissue-specific experiment, the seed dry-mass proportion showed no significant association with the measured whole-fruit oil content, whereas dry-mass-weighted reconstruction from seed and non-seed oil contents closely reproduced the directly measured whole-fruit oil values. The reconstructed and measured values were strongly correlated, with little mean bias and a low absolute error. Together, these results indicate that an accession-level variation in whole-fruit oil content reflects a tissue-specific oil concentration in addition to the relative contribution of the two tissue fractions. This interpretation reduces the likelihood that the whole-fruit differences observed here were driven primarily by variation in the seed-to-non-seed mass ratio.

The tissue-specific fatty acid contrasts also provide a clearer biological interpretation of the geographic patterns observed in the whole-fruit profiles. Differences in whole-fruit C16:0, C18:1, and C18:2 can arise from both the relative abundance of seed and non-seed tissues and their distinct fatty acid compositions. Accordingly, origin-level differences in whole-fruit fatty acid composition should not be interpreted as evidence for a single underlying biochemical mechanism. Changes in desaturation, triacylglycerol assembly, tissue development, and relative tissue contribution may all contribute to the observed composition [51,52,53,54]. Tissue-resolved measurements therefore provide a more appropriate basis for future mechanistic studies of oil accumulation in *S. paniculata*.

The present data also support a more cautious interpretation of germplasm value. Some sampled origins contained accessions with relatively high whole-fruit oil content or distinctive fatty acid profiles, but these measurements were obtained from field-grown trees in different environments. Environmental effects and genotype-by-environment interactions cannot be separated from accession effects in the current design. Therefore, the observed oil and fatty acid differences are best treated as characteristics of the sampled accessions under their source environments rather than as fixed breeding values. Replicated common-garden and multi-year measurements will be required before tissue-specific oil traits or whole-fruit composition can be used for stable donor classification or parent selection.

### 4.3. Genetic Structure and Tissue Measurements Refine the Interpretation of Relationships Between Quantitative Traits and Oil Content

Correlations calculated across all 30 accessions describe covariation in the sampled material, but their interpretation depends strongly on genetic structure. In the original pooled analysis, nine of the seventy-two relationships between quantitative traits and oil-related variables remained significant after multiple-testing correction. The strongest pooled relationships with whole-fruit oil content involved the infructescence width (IW), fruit transverse diameter (FTD), and fruit longitudinal diameter (FLD). However, geographic origin and genetic structure were associated with substantial variations among accessions, and pooled correlations can therefore combine differences among origins with covariation among accessions within origins [16,17,18,34].

This distinction became evident after adjustment. The coefficients relating IW, FTD, and FLD to whole-fruit oil content remained positive in both the origin-adjusted and genetic-adjusted analyses, but none of the 72 relationships retained significance after Benjamini–Hochberg correction within the corresponding adjusted test families. Thus, the persistence of coefficient direction should not be interpreted as statistical confirmation of these relationships. Instead, FLD, FTD, and IW should be regarded as candidate phenotypic variables whose association with whole-fruit oil content requires independent validation. The same caution applies to relationships between quantitative traits and individual fatty acids that showed similar coefficient directions across models but did not retain multiplicity-corrected support after adjustment.

The tissue-resolved analysis provides an additional layer of evidence because it separates the whole-fruit oil content into biologically distinct components. Among the 15 combinations evaluated, the positive relationship between IW and non-seed oil content was the only association that remained significant after Benjamini–Hochberg correction in the pooled, origin-adjusted, and genetic-adjusted analyses. In contrast, IW, FTD, and FLD showed no significant relationships with the seed dry-mass proportion or seed oil content in any of the three analytical models. These results indicate that the previously observed whole-fruit relationships were not simply explained by the variation in seed proportion or seed oil concentration and suggest that oil accumulation in non-seed tissues may contribute more directly to the association involving infructescence width.

Nevertheless, the IW and non-seed oil relationship remains observational. The current analysis does not establish a causal connection between infructescence morphology and lipid accumulation, nor does it demonstrate that IW can predict oil content reliably in independent material. Several other relationships involving measured or reconstructed whole-fruit oil content were significant in one or two of the tissue-specific adjusted models, but their support was less consistent across analytical frameworks. Accordingly, these associations are better treated as testable hypotheses rather than validated selection criteria.

For a practical evaluation of *S. paniculata* germplasm, direct measurement of oil content should therefore remain the primary basis for identifying high-oil accessions. Morphological traits such as IW, FTD, and FLD may be recorded as complementary phenotypes and prioritized for validation, but they should not yet be used as stand-alone pre-screening indicators. Replicated clonal common-garden trials, measurements across years, and independent accession sets are needed to determine the repeatability, predictive value, and environmental sensitivity of these relationships [6,7]. Such validation would also clarify whether the relatively stable IW and non-seed oil association reflects a reproducible biological relationship or a feature specific to the present set of 30 elite accessions.

### 4.4. Implications for Germplasm Conservation and Future Validation

The combined genetic, chemical, and quantitative trait data indicate that the sampled *S. paniculata* accessions capture complementary forms of variation, but the six geographic origins should not be interpreted as fully defined biological populations. Within this 30-accession panel, the Longshan group showed the highest values of several genetic diversity indices and also included accessions with relatively high whole-fruit oil content and C18:1 proportions. Daweishan included accessions with a high whole-fruit oil content, whereas the sampled Hengshan and Daoxian accessions showed relatively high MUFA and PUFA proportions, respectively. These contrasts identify useful variations within the present collection, but they should be interpreted as properties of the sampled accessions rather than as generalized characteristics of all germplasm from the corresponding regions.

For conservation, the current results support retaining accessions that represent different geographic origins, genetic positions, and oil-related phenotypes rather than prioritizing material solely on the basis of origin means. Core collection design can integrate geographic origin, genetic composition, and target phenotypes [55,56,57]. Accessions occupying distinctive positions in the PCoA or neighbor-joining tree, as well as accessions with unusual oil or fatty acid profiles, may therefore be useful for maintaining the breadth of variation represented in this panel. The relatively high diversity observed among the sampled Longshan accessions suggests that additional sampling from this region would be informative, but broader sampling is required before assigning conservation priority at the regional level.

The present data provide hypotheses for subsequent pre-breeding rather than validated parent recommendations. Direct measurements of whole-fruit and tissue-specific oil contents should remain the primary basis for evaluating oil-related performance. The tissue-specific reassessment identified a positive relationship between infructescence width and non-seed oil content that remained significant after both origin and genetic adjustments, making this relationship suitable for independent validation. By contrast, the original whole-fruit relationships involving the fruit transverse diameter, fruit longitudinal diameter, and infructescence width did not retain Benjamini–Hochberg significance after a structure adjustment within the 72-test families and should not yet be treated as selection criteria.

A major limitation is that the phenotypic and chemical measurements were obtained from field-grown trees originating from different environments. The present design therefore cannot separate genetic effects from environmental effects or genotype-by-environment interactions. Replicated clonal common-garden trials and multi-year measurements are needed to determine the repeatability of whole-fruit oil content, tissue-specific oil accumulation, fatty acid composition, and candidate morphology–oil relationships. Controlled crosses and progeny testing would become appropriate only after stable parental phenotypes have been confirmed under comparable environments. Larger and more geographically extensive collections would also improve estimates of genetic diversity and differentiation and provide a stronger basis for future association or genomic-prediction analyses.

Accordingly, the present study is best viewed as a resource characterization framework that identifies genetic and phenotypic contrasts requiring further validation. Its immediate value lies in documenting variations among the sampled elite accessions, identifying materials and traits that merit replicated evaluation, and providing a structure-aware basis for designing subsequent conservation and breeding experiments.

## 5. Conclusions

By integrating 6335 GBS-derived SNPs, whole-fruit and tissue-specific oil traits, fatty acid composition, and 12 quantitative traits, this study characterized genetic and phenotypic variations among 30 elite *Symplocos paniculata* accessions from six geographic origins in Hunan Province. Genetic differentiation among origins was accompanied by substantial within-origin heterogeneity, and the principal PCoA, diversity, AMOVA, and pairwise FST patterns remained stable after exclusion of the high-missingness accession, supporting the robustness of the main genetic conclusions within the sampled panel. Tissue-specific analysis showed that seed and non-seed tissues differed in both oil content and fatty acid composition, while dry-mass-weighted reconstruction closely reproduced measured whole-fruit oil content, indicating that whole-fruit variations reflect tissue-specific oil accumulation rather than tissue proportion alone. The original pooled relationships between fruit or infructescence traits and whole-fruit oil content did not retain Benjamini–Hochberg significance after adjustment for geographic origin or genetic structure; however, the relationship between infructescence width and non-seed oil content remained significant in the separate tissue-specific pooled, origin-adjusted, and genetic-adjusted analyses and therefore represents a candidate for independent validation rather than a validated selection criterion. Overall, the sampled accessions contain complementary genetic and oil-related variations, but their use in conservation and breeding should prioritize direct chemical measurements and broader germplasm representation, followed by replicated common-garden and multi-year validation before stable donor or parent assignments are made.

## Figures and Tables

**Figure 1:**
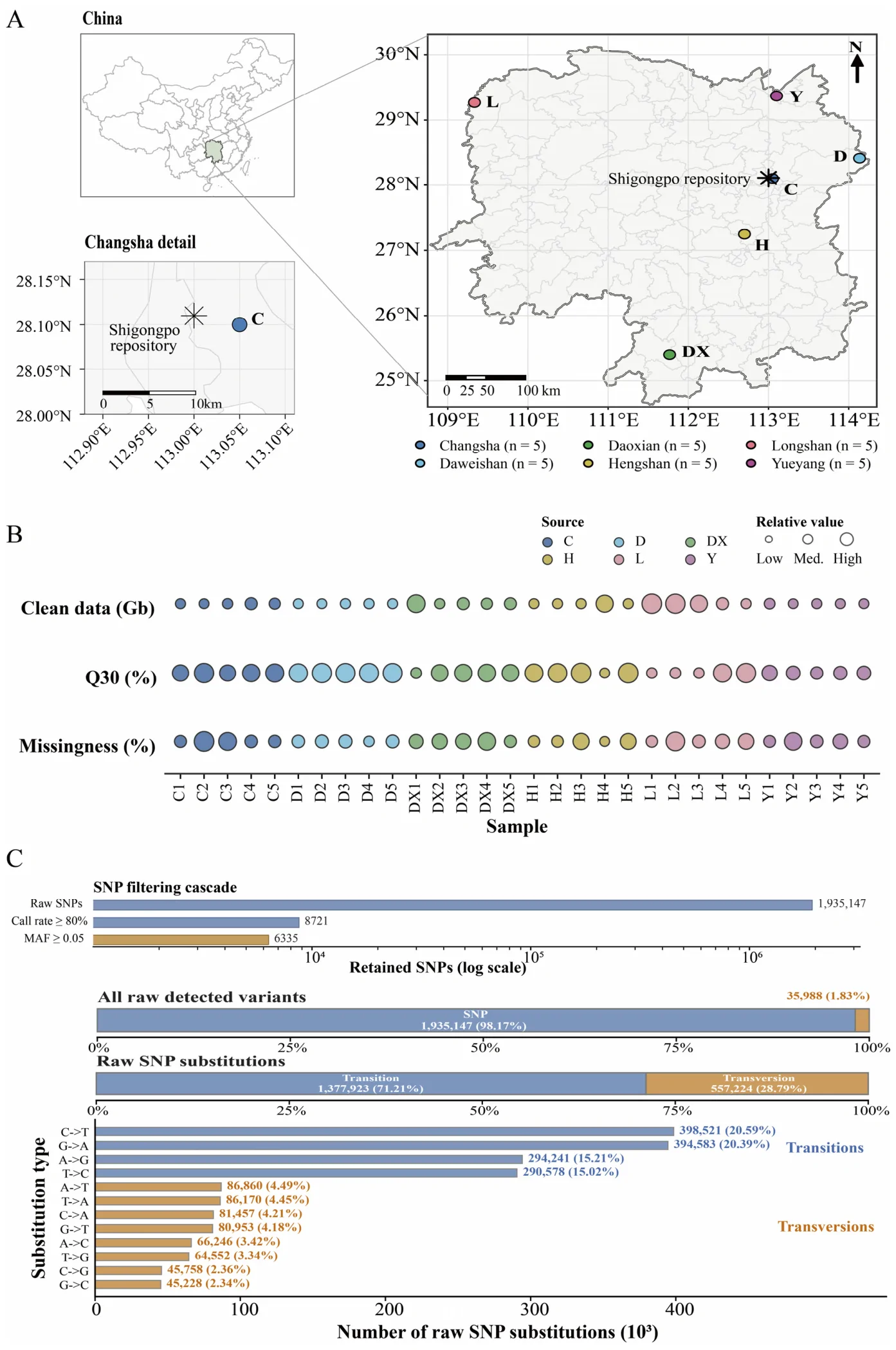
Sampling framework, GBS data quality, and variant filtering for 30 *S. paniculata* accessions. (**A**) Sampling localities for the six geographic origins in Hunan Province and the Shigongpo repository. (**B**) Accession-level clean-data volume, Q30, and genotype missingness; colors indicate geographic origin and point sizes indicate relative values. (**C**) SNP filtering, composition of raw detected variants, transition and transversion proportions, and the SNP substitution spectrum.

**Figure 2:**
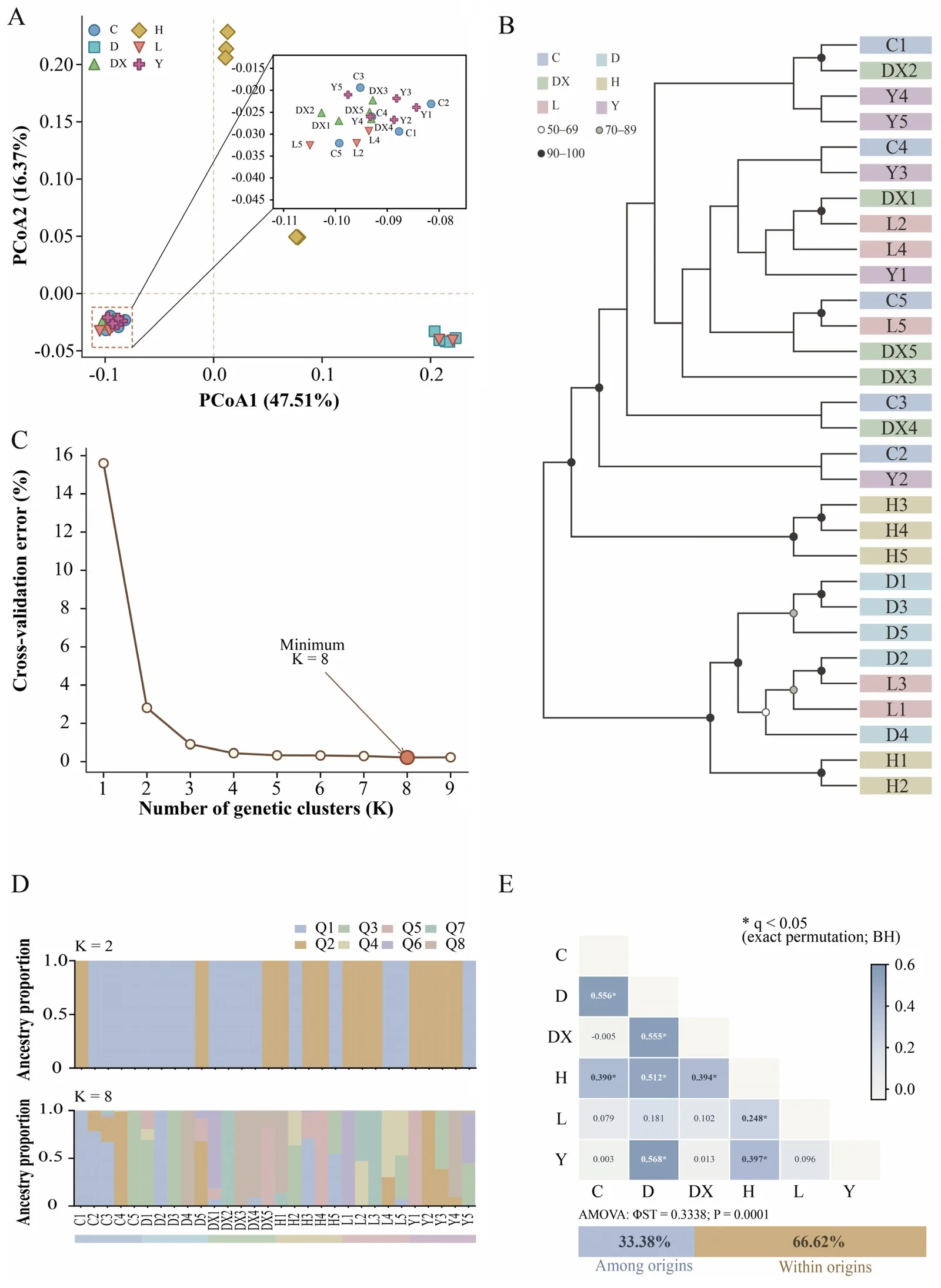
Genetic diversity, differentiation, and ancestry patterns of 30 *S. paniculata* accessions. (**A**) Cailliez-corrected principal coordinate analysis (PCoA) based on 6335 filtered SNPs; colors indicate geographic origin. (**B**) Neighbor-joining tree based on the same SNP dataset; terminal colors indicate geographic origin, and circle colors indicate internal-node support classes. (**C**) Archived ADMIXTURE cross-validation error for K = 1–9. (**D**) Ancestry proportions at K = 2 and K = 8. K = 2 illustrates coarse-scale ancestry composition, whereas K = 8, which corresponds to the minimum of the available cross-validation curve, is shown as an exploratory finer-scale representation rather than as a definitive number of genetic populations. (**E**) Pairwise Weir–Cockerham FST estimates among the six sampled geographic origins and AMOVA results; asterisks indicate Benjamini–Hochberg-adjusted q < 0.05.

**Figure 3:**
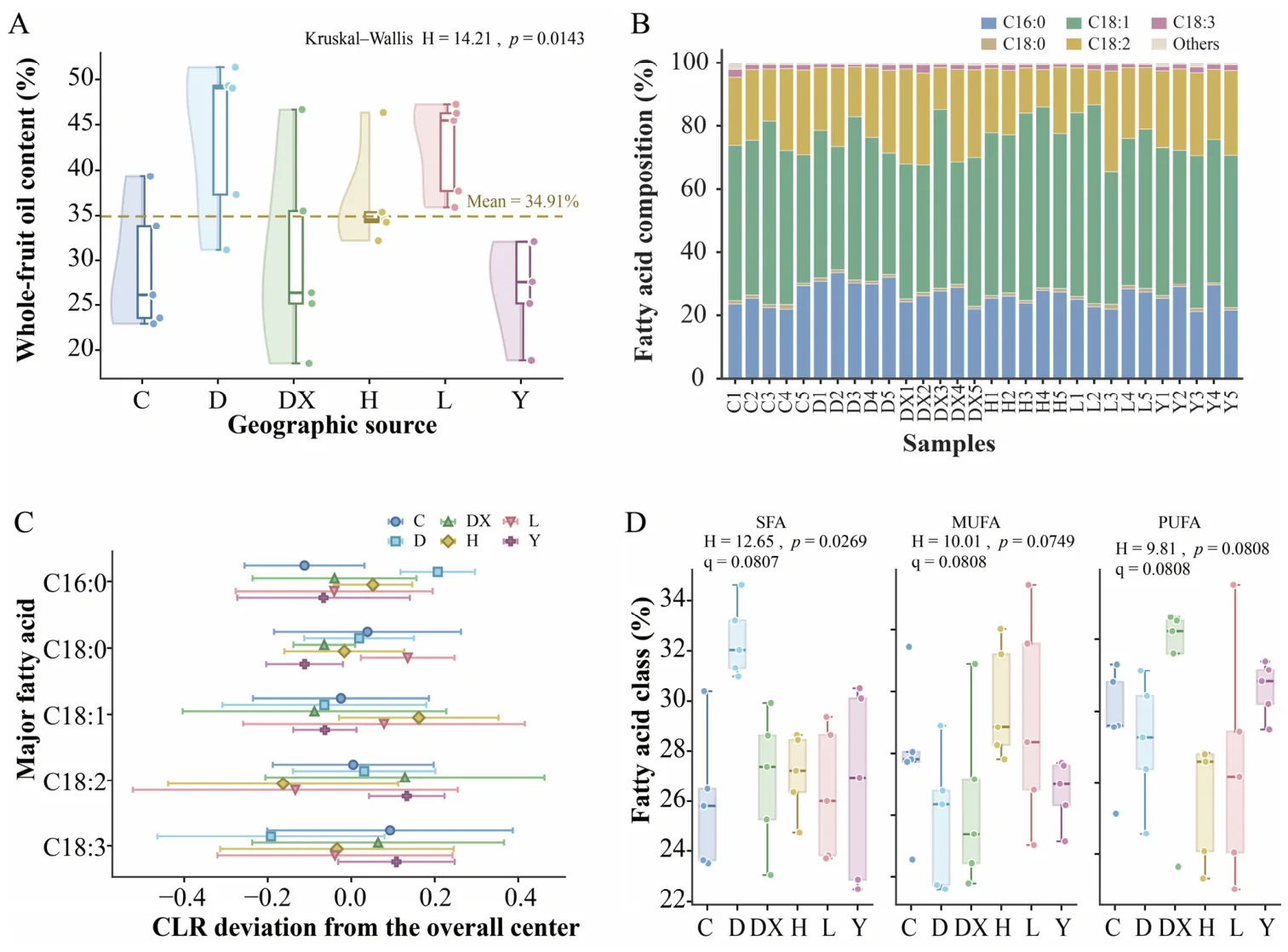
Variation in whole-fruit oil content and fatty acid composition among 30 *S. paniculata* accessions. (**A**) Whole-fruit oil content distributions by geographic origin; the dashed line indicates the overall mean. (**B**) Accession-level fatty acid composition. (**C**) Origin-level CLR deviations from the overall compositional center; points show origin means and horizontal bars show descriptive 95% intervals. (**D**) Distributions of saturated, monounsaturated, and polyunsaturated fatty acids among geographic origins; displayed H- and *p*-values are unadjusted Kruskal–Wallis results, and the corresponding q-values are Benjamini–Hochberg-adjusted results for the three-test family.

**Figure 4:**
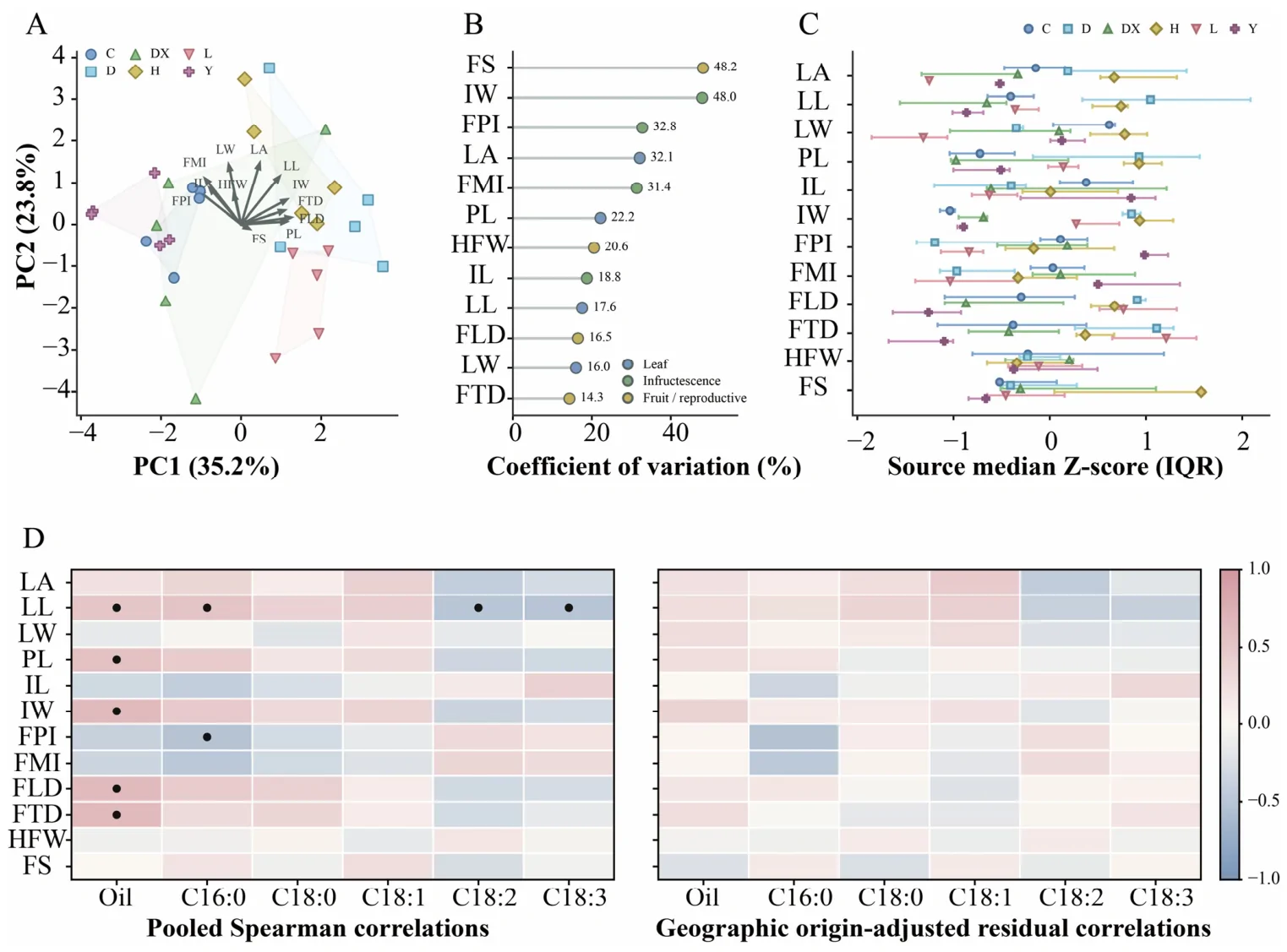
Quantitative trait variations and relationships with whole-fruit oil contents and fatty acid compositions. (**A**) Principal component biplot for 12 standardized quantitative traits; arrows represent trait-loading vectors, and translucent convex hulls show the observed ranges of accessions from each geographic origin in the first two principal-component dimensions. (**B**) Coefficients of variation for the 12 quantitative traits. (**C**) Median and interquartile ranges of Z scores for all 12 traits in each geographic origin. (**D**) Pooled Spearman correlations and geographic origin-adjusted residual correlations between the 12 quantitative traits and whole-fruit oil content or five fatty acid CLR values; black dots indicate pooled relationships with Benjamini–Hochberg-adjusted q < 0.05.

**Table 1:** Geographic origins and sampling information for the 30 *S. paniculata* accessions.

Origin	Locality	Longitude (°E)	Latitude (°N)	Elevation (m)	*n*	Sample ID
C	Changsha	113.05	28.1	78	5	10, 14, 15, 20, 25
D	Daweishan	114.15	28.417	1550	5	3, 19, 21, 29, 30
DX	Daoxian	111.767	25.4	100	5	5, 16, 17, 23, 28
H	Hengshan	112.7	27.25	650	5	11, 18, 22, 24, 26
L	Longshan	109.333	29.267	1402	5	1, 2, 6, 13, 27
Y	Yueyang	113.1	29.367	80	5	4, 7, 8, 9, 12

Note: Analysis Sample IDs 1–30 are used in the tables and supplementary data. Origin-coded Figure IDs are used when individual accessions are labeled in the main and supplementary figures; the one-to-one correspondence between the two identifier systems is provided in Supplementary Table S1. GBS, whole-fruit oil, fatty acid composition, and quantitative trait data were available for all 30 accessions.

**Table 2:** Origin-level genetic diversity of the 30 *S. paniculata* accessions based on 6335 filtered SNPs.

Origin	*n*	Loci Included	Ho	He	uHe	PIC
C	5	6233	0.041	0.138	0.156	0.111
D	5	5974	0.024	0.099	0.112	0.078
DX	5	6321	0.082	0.147	0.164	0.118
H	5	5792	0.040	0.153	0.172	0.119
L	5	6321	0.115	0.225	0.254	0.180
Y	5	5896	0.029	0.126	0.144	0.101

Note: A locus contributed to an origin-level estimate when at least three of the five accessions from that geographic origin had valid genotypes. Ho, observed heterozygosity; He, expected heterozygosity; uHe, unbiased expected heterozygosity; PIC, polymorphism information content. Values summarize the 30-accession reference dataset based on 6335 filtered SNPs. Geographic origins represent the sampling groups used in this study and should not be interpreted as fully defined biological populations.

**Table 3:** Descriptive statistics and geographic origin effects for whole-fruit oil content, five major fatty acids, three fatty acid classes, and twelve quantitative traits.

Variable	Unit	*n*	Mean ± SD	Range	CV (%)	H	*p*	q
Whole-fruit oil content	%	30	34.91 ± 9.49	18.54–51.35	27.18	14.213	0.0143	0.0143
C16:0	%	30	26.24 ± 3.41	21.06–33.54	13.00	12.814	0.0252	0.1259
C18:0	%	30	1.03 ± 0.17	0.80–1.60	16.84	7.568	0.1817	0.2271
C18:1	%	30	48.53 ± 6.56	38.47–63.16	13.52	10.012	0.0749	0.1835
C18:2	%	30	22.01 ± 5.64	10.97–31.71	25.63	8.974	0.1101	0.1835
C18:3	%	30	1.46 ± 0.43	0.76–2.65	29.21	5.526	0.3551	0.3551
SFA	%	30	27.28 ± 3.38	22.12–34.47	12.38	12.649	0.0269	0.0807
MUFA	%	30	48.53 ± 6.56	38.47–63.17	13.52	10.012	0.0749	0.0808
PUFA	%	30	23.47 ± 5.84	12.57–33.78	24.89	9.810	0.0808	0.0808
LA	cm^2^	30	18.29 ± 5.88	10.47–30.78	32.14	12.923	0.0241	0.0413
LL	cm	30	6.63 ± 1.16	4.79–9.04	17.55	16.109	0.0065	0.0196
LW	cm	30	3.55 ± 0.57	2.52–4.83	16.00	14.161	0.0146	0.0351
PL	cm	30	0.58 ± 0.13	0.36–0.92	22.20	13.457	0.0195	0.0389
IL	cm	30	9.29 ± 1.74	5.13–13.50	18.76	7.539	0.1835	0.2002
IW	cm	30	5.48 ± 2.63	1.73–10.44	47.97	19.328	0.0017	0.0104
FPI	count	30	29.60 ± 9.71	14.00–48.00	32.80	10.938	0.0526	0.0789
FMI	g	30	3.10 ± 0.97	1.60–5.14	31.39	9.330	0.0966	0.1288
FLD	mm	30	7.31 ± 1.20	5.14–9.56	16.48	19.235	0.0017	0.0104
FTD	mm	30	6.15 ± 0.88	4.68–7.65	14.32	16.405	0.0058	0.0196
HFW	g	30	8.51 ± 1.75	4.46–14.22	20.56	0.272	0.9981	0.9981
FS	%	30	38.96 ± 18.77	12.56–86.57	48.18	7.741	0.1711	0.2002

Note: H and *p* are from the Kruskal–Wallis tests across the six geographic origins. Benjamini–Hochberg adjustment was applied separately to the single-oil content test, the five-fatty-acid family, the three-fatty-acid-class family, and the twelve-quantitative-trait family. CV, coefficient of variation.

## Data Availability

The data supporting the findings of this study are available within the article and its Supplementary Materials. Additional data are available from the corresponding author upon reasonable request.

## Supplementary Materials

The following supporting information can be downloaded at: https://www.mdpi.com/article/10.3390/biology15191702/s1, Figure S1: Accession-level distributions of fatty-acid classes among the six geographic origins. (A) Saturated fatty acids (SFA), calculated as C16:0 + C18:0; (B) monounsaturated fatty acids (MUFA), represented by C18:1; and (C) polyunsaturated fatty acids (PUFA), calculated as C18:2 + C18:3. Points show individual accessions, horizontal lines show origin means, and colors and symbols identify geographic origins. Figure S2. Geographic origin variations in the 12 quantitative traits: (A) leaf area (LA); (B) leaf length (LL); (C) leaf width (LW); (D) petiole length (PL); (E) infructescence length (IL); (F) infructescence width (IW); (G) fruits per infructescence (FPI); (H) fruit mass per infructescence (FMI); (I) fruit longitudinal diameter (FLD); (J) fruit transverse diameter (FTD); (K) 100-fruit fresh weight (HFW); and (L) fruit set (FS). Points represent accessions, boxes summarize within-origin distributions, H- and *p*-values are from Kruskal–Wallis tests, and q is the Benjamini–Hochberg-adjusted value for the 12-test family. Figure S3. Relationships between the 12 quantitative traits and whole-fruit oil content. (A) Pooled Spearman correlations across all 30 accessions. (B) Geographic origin-adjusted residual correlations, calculated as Pearson correlations between residuals of rank-transformed variables after regression on geographic origin indicators. Points show correlation coefficients, horizontal bars show 95% origin-stratified bootstrap confidence intervals, and background shading distinguishes leaf, infructescence, and fruit/reproductive traits. Figure S4. Sensitivity of SNP retention and PCoA to call-rate and minor-allele-frequency (MAF) thresholds. (A) Number of SNPs retained under nine combinations of call-rate and MAF thresholds. (B) Correlations between the corresponding genetic distance matrices and the reference matrix based on call rate ≥ 80% and MAF ≥ 0.05. (C–E) Cailliez-corrected PCoA under liberal, reference, and stringent filtering schemes, respectively; colors and symbols indicate geographic origins. Figure S5. Results of the additional tissue-specific experiment: dry-mass contribution, oil content, whole-fruit reconstruction, and fatty acid composition across 30 *S. paniculata* accessions. (A) Seed and non-seed dry-mass proportions. (B) Paired seed and non-seed oil contents; the *p*-value is from the paired Wilcoxon signed-rank test. (C) Directly measured versus dry-mass-weighted reconstructed whole-fruit oil content; colors indicate geographic origin. (D) Mean relative abundances of C16:0, C18:0, C18:1, C18:2, and C18:3 in seed and non-seed oils. Figure S6. Tissue-specific reassessment of relationships between fruit longitudinal diameter (FLD), fruit transverse diameter (FTD), infructescence width (IW), and five tissue- or oil-related variables. (A) Pooled Spearman correlations. (B) Geographic origin-adjusted residual correlations. (C) Genetic-adjusted residual correlations. (D) Pooled relationship between IW and non-seed oil content. Black dots in panels A–C indicate Benjamini–Hochberg-adjusted q < 0.05 within the corresponding 15-test family. Figure S7. Individual-missingness sensitivity analysis after exclusion of the high-missingness accession L5. Cailliez-corrected PCoA was recalculated for 29 accessions after reapplying site call rate ≥ 80% and MAF ≥ 0.05, yielding 5672 SNPs. PCoA1 and PCoA2 explained 46.52% and 16.77% of the positive-eigenvalue variation, respectively; colors and symbols indicate geographic origins. Supplementary tables: Table S1: Accession identifiers and geographic origins; Table S2: Per-accession GBS sequencing and genotype-quality metrics; Table S3: Variant overview, substitution spectrum, and SNP filtering; Table S4: Pairwise genetic differentiation and AMOVA; Table S5: Geographic origin summaries and Dunn comparisons for whole-fruit oil contents; Table S6: Accession-level oil, fatty acid, and quantitative trait data; Table S7: Complete results for associations between quantitative traits and oil-related variables; Table S8: Reproducibility parameters and analysis manifest; Table S9: Accession-level seed and non-seed dry-mass proportions, tissue oil contents, tissue fatty acid compositions, and SFA, MUFA, and PUFA values; Table S10: Complete results for the 15 tissue-specific morphology-by-oil associations in pooled, geographic origin-adjusted, and genetic-adjusted analyses; Table S11: Comparison of the 30-accession reference dataset and 29-accession individual-missingness sensitivity dataset, including SNP retention, origin-level diversity, AMOVA, and pairwise FST results.
